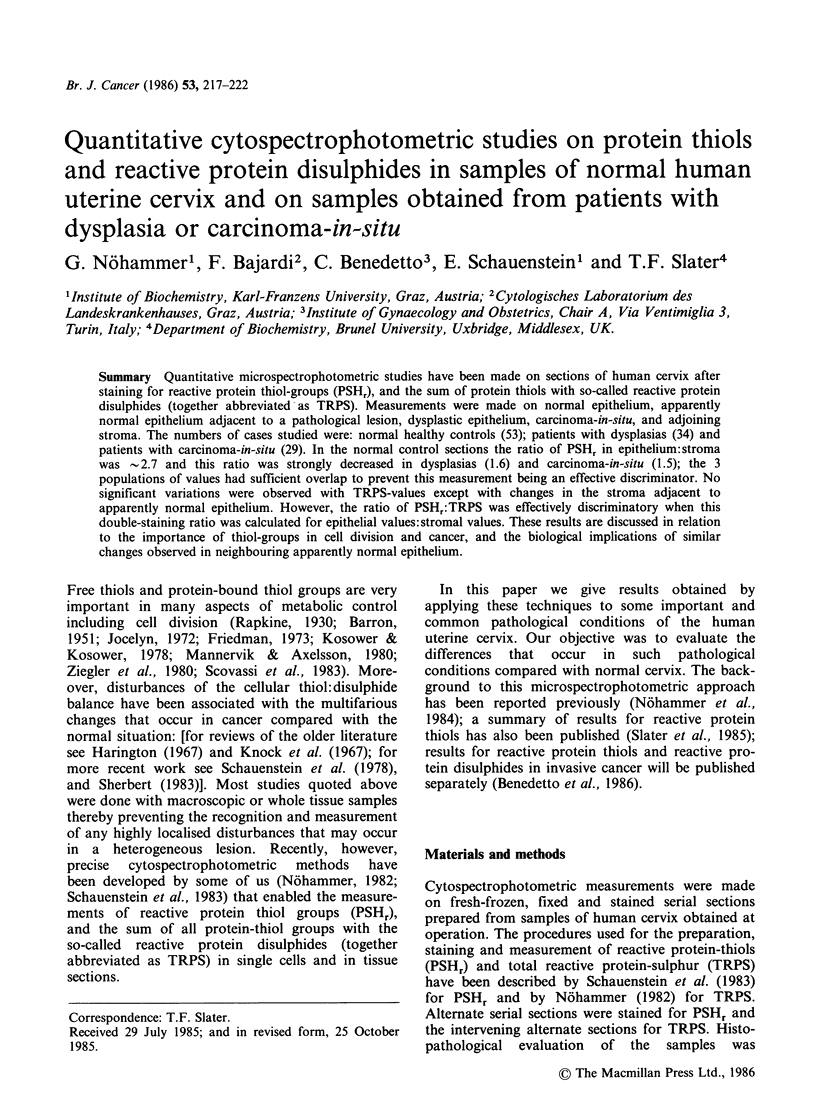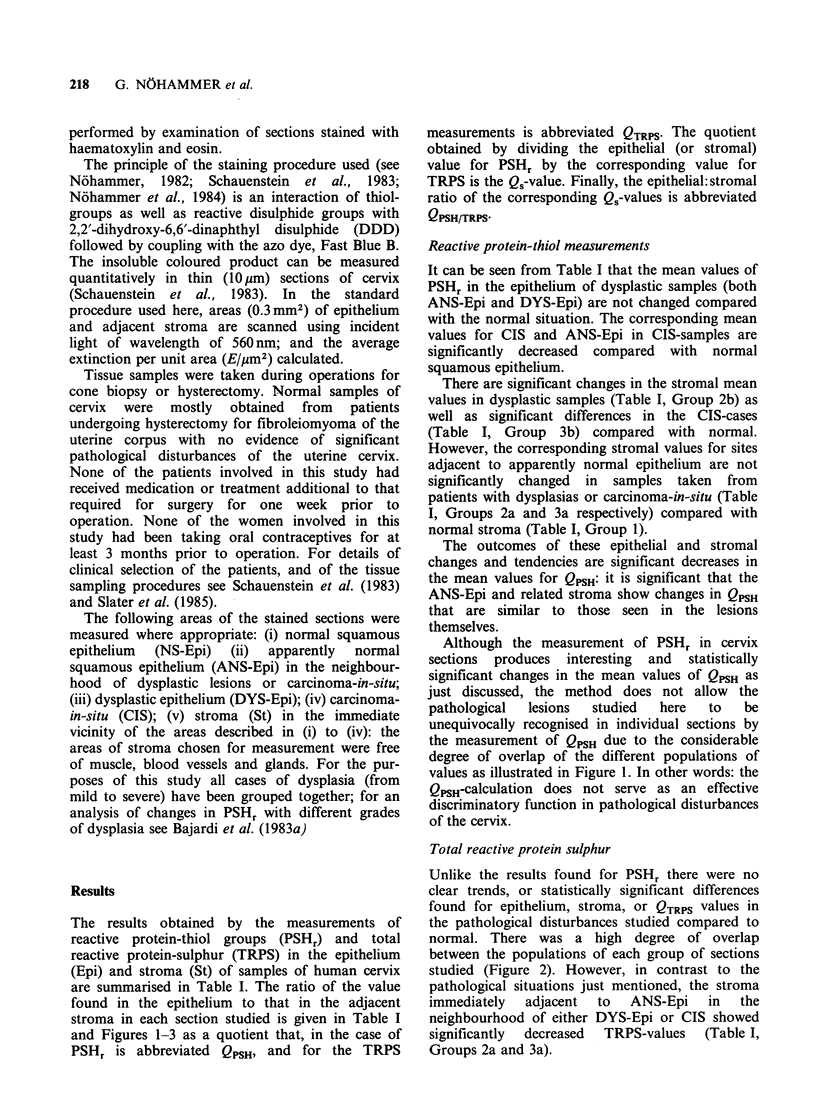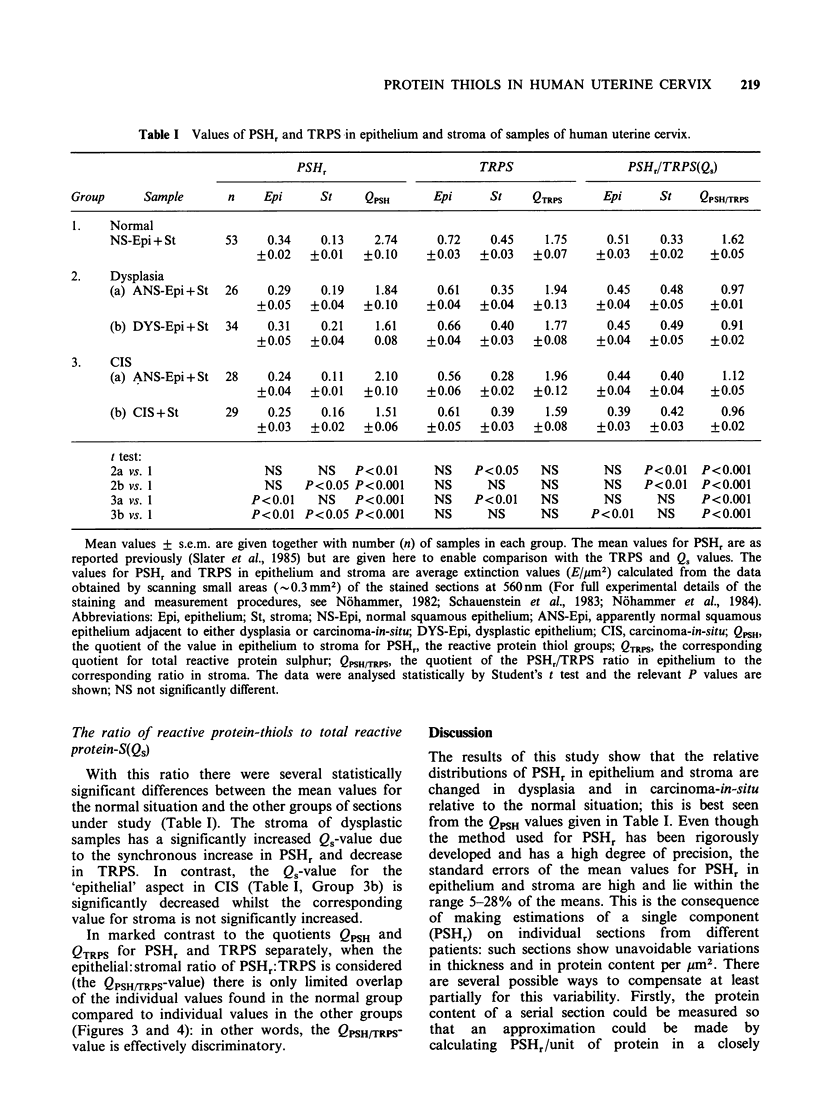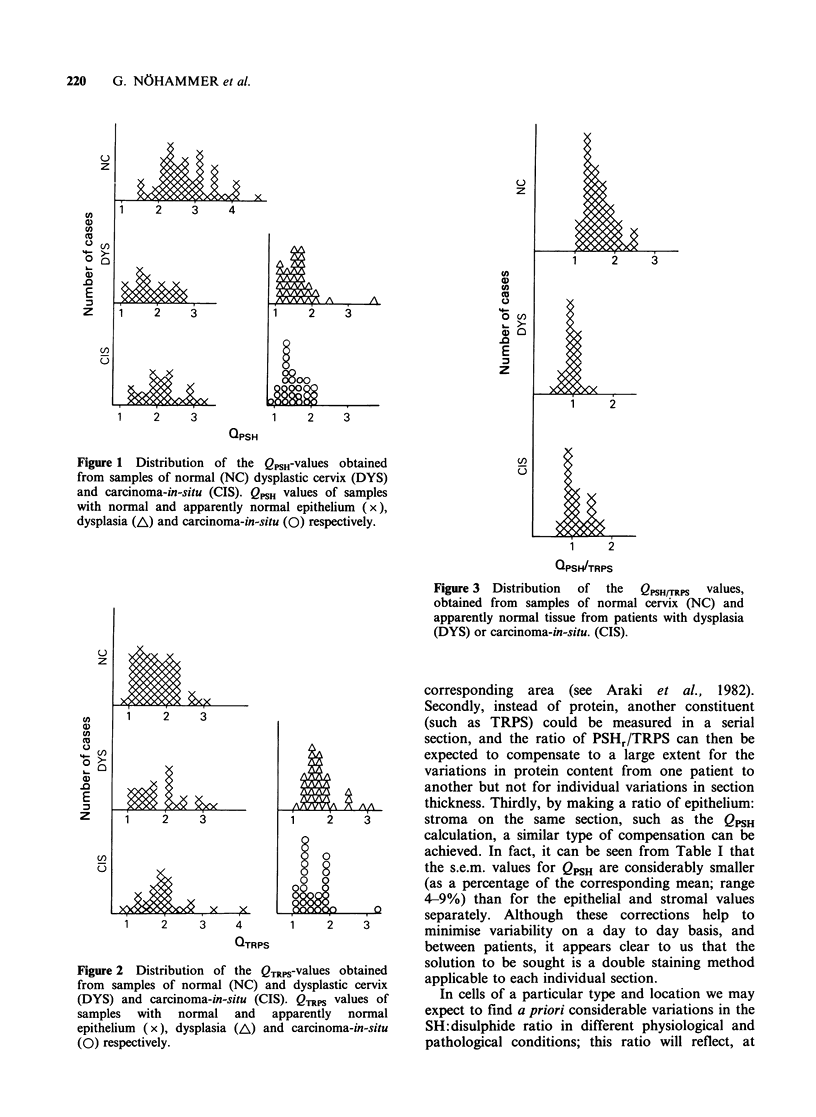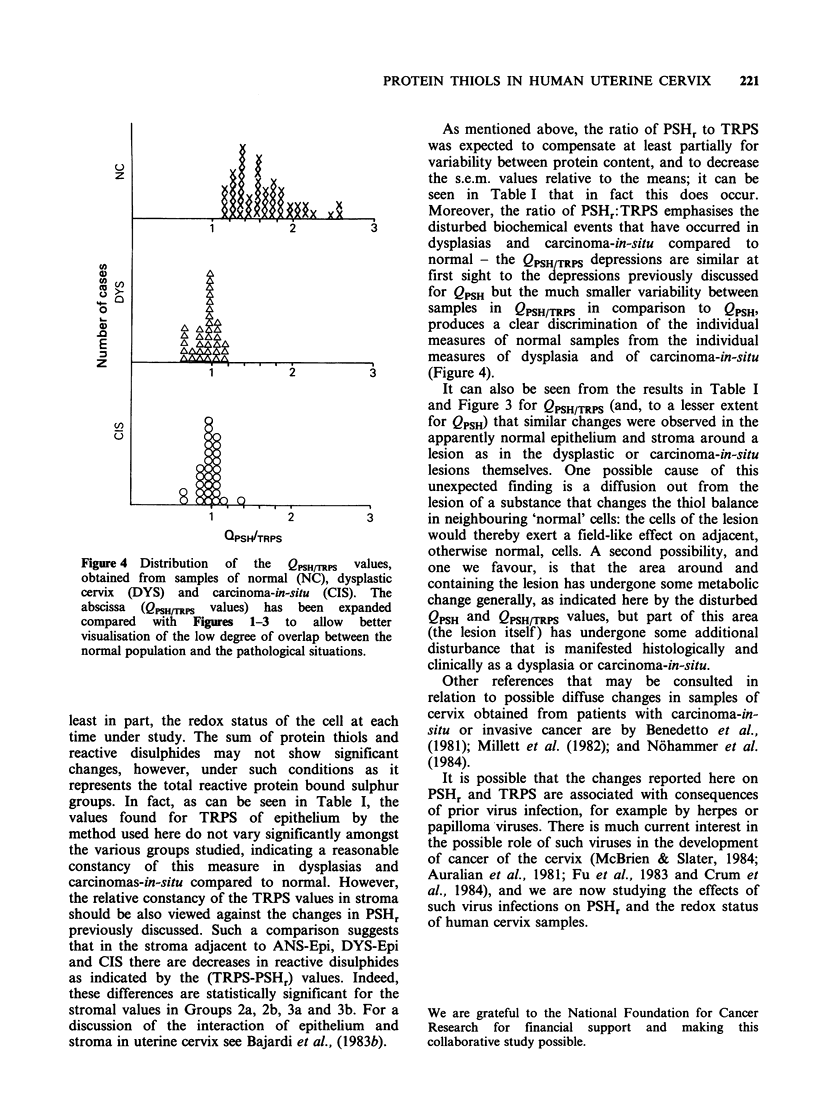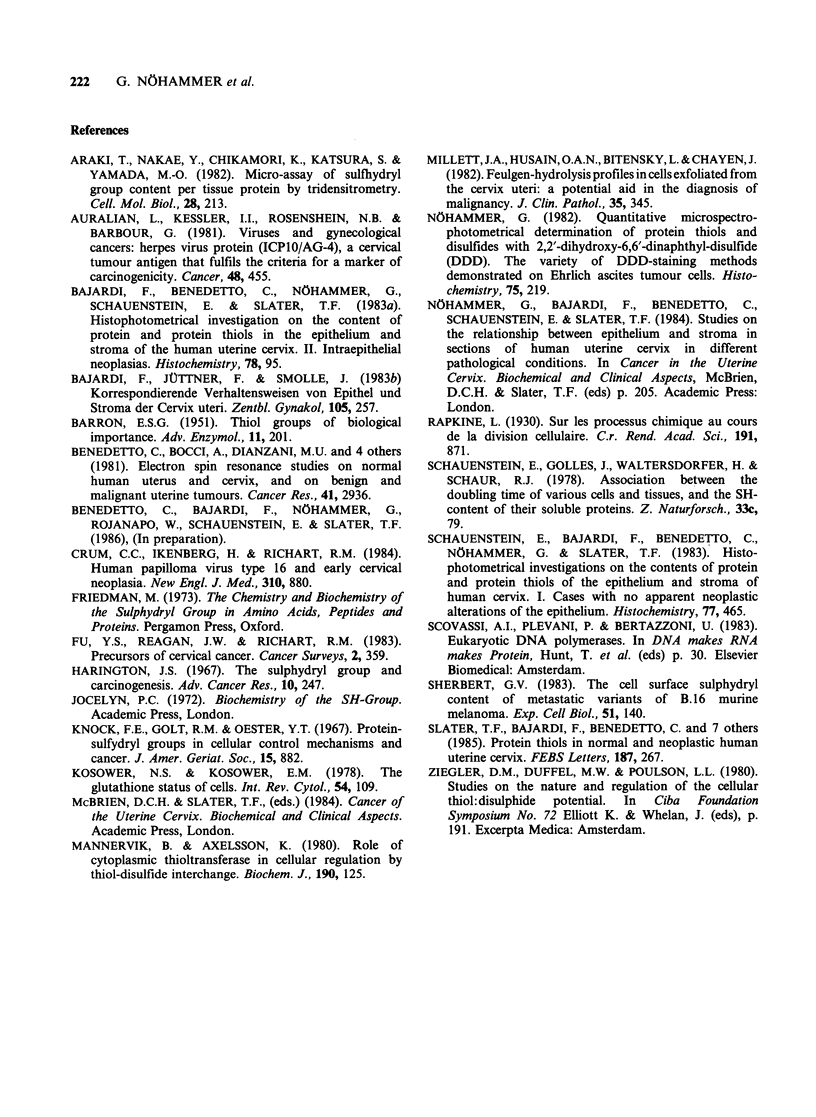# Quantitative cytospectrophotometric studies on protein thiols and reactive protein disulphides in samples of normal human uterine cervix and on samples obtained from patients with dysplasia or carcinoma-in-situ.

**DOI:** 10.1038/bjc.1986.38

**Published:** 1986-02

**Authors:** G. Nöhammer, F. Bajardi, C. Benedetto, E. Schauenstein, T. F. Slater

## Abstract

Quantitative microspectophotometric studies have been made on sections of human cervix after staining for reactive protein thiol-groups (PSHr), and the sum of protein thiols with so-called reactive protein disulphides (together abbreviated as TRPS). Measurements were made on normal epithelium, apparently normal epithelium adjacent to a pathological lesion, dysplastic epithelium, carcinoma-in-situ, and adjoining stroma. The numbers of cases studied were: normal healthy controls (53); patients with dysplasias (34) and patients with carcinoma-in-situ (29). In the normal control sections the ratio of PSHr in epithelium:stroma was approximately 2.7 and this ratio was strongly decreased in dysplasias (1.6) and carcinoma-in-situ (1.5); the 3 populations of values had sufficient overlap to prevent this measurement being an effective discriminator. No significant variations were observed with TRPS-values except with changes in the stroma adjacent to apparently normal epithelium. However, the ratio of PSHr:TRPS was effectively discriminatory when this double-staining ratio was calculated for epithelial values:stromal values. These results are discussed in relation to the importance of thiol-groups in cell division and cancer, and the biological implications of similar changes observed in neighbouring apparently normal epithelium.


*Br. J. Cancer* (1986) **53**, 217–222

### Quantitative cytospectrophotometric studies on protein thiols and reactive protein disulphides in samples of normal human uterine cervix and on samples obtained from patients with dysplasia or carcinoma-*in-situ*

G. Nöhammer[1], F. Bajardi[2], C. Benedetto[3], E. Schauenstein[1] and T.F. Slater[4]

[1]*Institute of Biochemistry, Karl-Franzens University, Graz, Austria;* [2]*Cytologisches Laboratorium des Landeskrankenhauses, Graz, Austria;* [3]*Institute of Gynaecology and Obstetrics, Chair A, Via Ventimiglia 3, Turin, Italy;* [4]*Department of Biochemistry, Brunel University, Uxbridge, Middlesex, UK.*

**Summary** Quantitative microspectrophotometric studies have been made on sections of human cervix after staining for reactive protein thiol-groups ($PSH_r$), and the sum of protein thiols with so-called reactive protein disulphides (together abbreviated as TRPS). Measurements were made on normal epithelium, apparently normal epithelium adjacent to a pathological lesion, dysplastic epithelium, carcinoma-*in-situ*, and adjoining stroma. The numbers of cases studied were: normal healthy controls (53); patients with dysplasias (34) and patients with carcinoma-*in-situ* (29). In the normal control sections the ratio of $PSH_r$ in epithelium:stroma was ~2.7 and this ratio was strongly decreased in dysplasias (1.6) and carcinoma-*in-situ* (1.5); the 3 populations of values had sufficient overlap to prevent this measurement being an effective discriminator. No significant variations were observed with TRPS-values except with changes in the stroma adjacent to apparently normal epithelium. However, the ratio of $PSH_r$:TRPS was effectively discriminatory when this double-staining ratio was calculated for epithelial values:stromal values. These results are discussed in relation to the importance of thiol-groups in cell division and cancer, and the biological implications of similar changes observed in neighbouring apparently normal epithelium.

Free thiols and protein-bound thiol groups are very important in many aspects of metabolic control including cell division (Rapkine, 1930; Barron, 1951; Jocelyn, 1972; Friedman, 1973; Kosower & Kosower, 1978; Mannervik & Axelsson, 1980; Ziegler *et al.*, 1980; Scovassi *et al.*, 1983). Moreover, disturbances of the cellular thiol:disulphide balance have been associated with the multifarious changes that occur in cancer compared with the normal situation: [for reviews of the older literature see Harington (1967) and Knock *et al.* (1967); for more recent work see Schauenstein *et al.* (1978), and Sherbert (1983)]. Most studies quoted above were done with macroscopic or whole tissue samples thereby preventing the recognition and measurement of any highly localised disturbances that may occur in a heterogeneous lesion. Recently, however, precise cytospectrophotometric methods have been developed by some of us (Nöhammer, 1982; Schauenstein *et al.*, 1983) that enabled the measurements of reactive protein thiol groups ($PSH_r$), and the sum of all protein-thiol groups with the so-called reactive protein disulphides (together abbreviated as TRPS) in single cells and in tissue sections.

Correspondence: T.F. Slater.
Received 29 July 1985; and in revised form, 25 October 1985.

In this paper we give results obtained by applying these techniques to some important and common pathological conditions of the human uterine cervix. Our objective was to evaluate the differences that occur in such pathological conditions compared with normal cervix. The background to this microspectrophotometric approach has been reported previously (Nöhammer *et al.*, 1984); a summary of results for reactive protein thiols has also been published (Slater *et al.*, 1985); results for reactive protein thiols and reactive protein disulphides in invasive cancer will be published separately (Benedetto *et al.*, 1986).

### Materials and methods

Cytospectrophotometric measurements were made on fresh-frozen, fixed and stained serial sections prepared from samples of human cervix obtained at operation. The procedures used for the preparation, staining and measurement of reactive protein-thiols ($PSH_r$) and total reactive protein-sulphur (TRPS) have been described by Schauenstein *et al.* (1983) for $PSH_r$ and by Nöhammer (1982) for TRPS. Alternate serial sections were stained for $PSH_r$ and the intervening alternate sections for TRPS. Histopathological evaluation of the samples was

© The Macmillan Press Ltd., 1986

performed by examination of sections stained with haematoxylin and eosin.

The principle of the staining procedure used (see Nöhammer, 1982; Schauenstein *et al.*, 1983; Nöhammer *et al.*, 1984) is an interaction of thiol-groups as well as reactive disulphide groups with 2,2′-dihydroxy-6,6′-dinaphthyl disulphide (DDD) followed by coupling with the azo dye, Fast Blue B. The insoluble coloured product can be measured quantitatively in thin (10 $\mu$m) sections of cervix (Schauenstein *et al.*, 1983). In the standard procedure used here, areas (0.3 $mm^2$) of epithelium and adjacent stroma are scanned using incident light of wavelength of 560 nm; and the average extinction per unit area ($E/\mu m^2$) calculated.

Tissue samples were taken during operations for cone biopsy or hysterectomy. Normal samples of cervix were mostly obtained from patients undergoing hysterectomy for fibroleiomyoma of the uterine corpus with no evidence of significant pathological disturbances of the uterine cervix. None of the patients involved in this study had received medication or treatment additional to that required for surgery for one week prior to operation. None of the women involved in this study had been taking oral contraceptives for at least 3 months prior to operation. For details of clinical selection of the patients, and of the tissue sampling procedures see Schauenstein *et al.* (1983) and Slater *et al.* (1985).

The following areas of the stained sections were measured where appropriate: (i) normal squamous epithelium (NS-Epi) (ii) apparently normal squamous epithelium (ANS-Epi) in the neighbourhood of dysplastic lesions or carcinoma-*in-situ*; (iii) dysplastic epithelium (DYS-Epi); (iv) carcinoma-*in-situ* (CIS); (v) stroma (St) in the immediate vicinity of the areas described in (i) to (iv): the areas of stroma chosen for measurement were free of muscle, blood vessels and glands. For the purposes of this study all cases of dysplasia (from mild to severe) have been grouped together; for an analysis of changes in $PSH_r$ with different grades of dysplasia see Bajardi *et al.* (1983*a)*

### Results

The results obtained by the measurements of reactive protein-thiol groups ($PSH_r$) and total reactive protein-sulphur (TRPS) in the epithelium (Epi) and stroma (St) of samples of human cervix are summarised in Table I. The ratio of the value found in the epithelium to that in the adjacent stroma in each section studied is given in Table I and Figures 1–3 as a quotient that, in the case of $PSH_r$ is abbreviated $Q_{PSH}$, and for the TRPS measurements is abbreviated $Q_{TRPS}$. The quotient obtained by dividing the epithelial (or stromal) value for $PSH_r$ by the corresponding value for TRPS is the $Q_s$-value. Finally, the epithelial:stromal ratio of the corresponding $Q_s$-values is abbreviated $Q_{PSH/TRPS}$.

### *Reactive protein-thiol measurements*

It can be seen from Table I that the mean values of $PSH_r$ in the epithelium of dysplastic samples (both ANS-Epi and DYS-Epi) are not changed compared with the normal situation. The corresponding mean values for CIS and ANS-Epi in CIS-samples are significantly decreased compared with normal squamous epithelium.

There are significant changes in the stromal mean values in dysplastic samples (Table I, Group 2b) as well as significant differences in the CIS-cases (Table I, Group 3b) compared with normal. However, the corresponding stromal values for sites adjacent to apparently normal epithelium are not significantly changed in samples taken from patients with dysplasias or carcinoma-*in-situ* (Table I, Groups 2a and 3a respectively) compared with normal stroma (Table I, Group 1).

The outcomes of these epithelial and stromal changes and tendencies are significant decreases in the mean values for $Q_{PSH}$: it is significant that the ANS-Epi and related stroma show changes in $Q_{PSH}$ that are similar to those seen in the lesions themselves.

Although the measurement of $PSH_r$ in cervix sections produces interesting and statistically significant changes in the mean values of $Q_{PSH}$ as just discussed, the method does not allow the pathological lesions studied here to be unequivocally recognised in individual sections by the measurement of $Q_{PSH}$ due to the considerable degree of overlap of the different populations of values as illustrated in Figure 1. In other words: the $Q_{PSH}$-calculation does not serve as an effective discriminatory function in pathological disturbances of the cervix.

### *Total reactive protein sulphur*

Unlike the results found for $PSH_r$ there were no clear trends, or statistically significant differences found for epithelium, stroma, or $Q_{TRPS}$ values in the pathological disturbances studied compared to normal. There was a high degree of overlap between the populations of each group of sections studied (Figure 2). However, in contrast to the pathological situations just mentioned, the stroma immediately adjacent to ANS-Epi in the neighbourhood of either DYS-Epi or CIS showed significantly decreased TRPS-values (Table I, Groups 2a and 3a).

**Table I** Values of $PSH_r$ and TRPS in epithelium and stroma of samples of human uterine cervix.

| | | | *$PSH_r$* | | | *TRPS* | | | *$PSH_r/TRPS(Q_s)$* | | |
|---|---|---|---|---|---|---|---|---|---|---|---|
| *Group* | *Sample* | *n* | *Epi* | *St* | *$Q_{PSH}$* | *Epi* | *St* | *$Q_{TRPS}$* | *Epi* | *St* | *$Q_{PSH/TRPS}$* |
| 1. | Normal | | | | | | | | | | |
| | NS-Epi+St | 53 | 0.34 ±0.02 | 0.13 ±0.01 | 2.74 ±0.10 | 0.72 ±0.03 | 0.45 ±0.03 | 1.75 ±0.07 | 0.51 ±0.03 | 0.33 ±0.02 | 1.62 ±0.05 |
| 2. | Dysplasia | | | | | | | | | | |
| | (a) ANS-Epi+St | 26 | 0.29 ±0.05 | 0.19 ±0.04 | 1.84 ±0.10 | 0.61 ±0.04 | 0.35 ±0.04 | 1.94 ±0.13 | 0.45 ±0.04 | 0.48 ±0.05 | 0.97 ±0.01 |
| | (b) DYS-Epi+St | 34 | 0.31 ±0.05 | 0.21 ±0.04 | 1.61 0.08 | 0.66 ±0.04 | 0.40 ±0.03 | 1.77 ±0.08 | 0.45 ±0.04 | 0.49 ±0.05 | 0.91 ±0.02 |
| 3. | CIS | | | | | | | | | | |
| | (a) ANS-Epi+St | 28 | 0.24 ±0.04 | 0.11 ±0.01 | 2.10 ±0.10 | 0.56 ±0.06 | 0.28 ±0.02 | 1.96 ±0.12 | 0.44 ±0.04 | 0.40 ±0.04 | 1.12 ±0.05 |
| | (b) CIS+St | 29 | 0.25 ±0.03 | 0.16 ±0.02 | 1.51 ±0.06 | 0.61 ±0.05 | 0.39 ±0.03 | 1.59 ±0.08 | 0.39 ±0.03 | 0.42 ±0.03 | 0.96 ±0.02 |
| | *t* test: | | | | | | | | | | |
| | 2a *vs.* 1 | | NS | NS | $P<0.01$ | NS | $P<0.05$ | NS | NS | $P<0.01$ | $P<0.001$ |
| | 2b *vs.* 1 | | NS | $P<0.05$ | $P<0.001$ | NS | NS | NS | NS | $P<0.01$ | $P<0.001$ |
| | 3a *vs.* 1 | | $P<0.01$ | NS | $P<0.001$ | NS | $P<0.01$ | NS | NS | NS | $P<0.001$ |
| | 3b *vs.* 1 | | $P<0.01$ | $P<0.05$ | $P<0.001$ | NS | NS | NS | $P<0.01$ | NS | $P<0.001$ |

Mean values ± s.e.m. are given together with number (*n*) of samples in each group. The mean values for $PSH_r$ are as reported previously (Slater *et al.*, 1985) but are given here to enable comparison with the TRPS and $Q_s$ values. The values for $PSH_r$ and TRPS in epithelium and stroma are average extinction values ($E/\mu m^2$) calculated from the data obtained by scanning small areas (~0.3 mm²) of the stained sections at 560 nm (For full experimental details of the staining and measurement procedures, see Nöhammer, 1982; Schauenstein *et al.*, 1983; Nöhammer *et al.*, 1984). Abbreviations: Epi, epithelium; St, stroma; NS-Epi, normal squamous epithelium; ANS-Epi, apparently normal squamous epithelium adjacent to either dysplasia or carcinoma-*in-situ*; DYS-Epi, dysplastic epithelium; CIS, carcinoma-*in-situ*; $Q_{PSH}$, the quotient of the value in epithelium to stroma for $PSH_r$, the reactive protein thiol groups; $Q_{TRPS}$, the corresponding quotient for total reactive protein sulphur; $Q_{PSH/TRPS}$, the quotient of the $PSH_r$/TRPS ratio in epithelium to the corresponding ratio in stroma. The data were analysed statistically by Student's *t* test and the relevant *P* values are shown; NS not significantly different.

### *The ratio of reactive protein-thiols to total reactive protein-S($Q_S$)*

With this ratio there were several statistically significant differences between the mean values for the normal situation and the other groups of sections under study (Table I). The stroma of dysplastic samples has a significantly increased $Q_s$-value due to the synchronous increase in $PSH_r$ and decrease in TRPS. In contrast, the $Q_s$-value for the 'epithelial' aspect in CIS (Table I, Group 3b) is significantly decreased whilst the corresponding value for stroma is not significantly increased.

In marked contrast to the quotients $Q_{PSH}$ and $Q_{TRPS}$ for $PSH_r$ and TRPS separately, when the epithelial:stromal ratio of $PSH_r$:TRPS is considered (the $Q_{PSH/TRPS}$-value) there is only limited overlap of the individual values found in the normal group compared to individual values in the other groups (Figures 3 and 4): in other words, the $Q_{PSH/TRPS}$-value is effectively discriminatory.

### Discussion

The results of this study show that the relative distributions of $PSH_r$ in epithelium and stroma are changed in dysplasia and in carcinoma-*in-situ* relative to the normal situation; this is best seen from the $Q_{PSH}$ values given in Table I. Even though the method used for $PSH_r$ has been rigorously developed and has a high degree of precision, the standard errors of the mean values for $PSH_r$ in epithelium and stroma are high and lie within the range 5–28% of the means. This is the consequence of making estimations of a single component ($PSH_r$) on individual sections from different patients: such sections show unavoidable variations in thickness and in protein content per $\mu m^2$. There are several possible ways to compensate at least partially for this variability. Firstly, the protein content of a serial section could be measured so that an approximation could be made by calculating $PSH_r$/unit of protein in a closely

**Figure 1** Distribution of the $Q_{PSH}$-values obtained from samples of normal (NC) dysplastic cervix (DYS) and carcinoma-*in-situ* (CIS). $Q_{PSH}$ values of samples with normal and apparently normal epithelium (×), dysplasia (△) and carcinoma-*in-situ* (○) respectively.

**Figure 2** Distribution of the $Q_{TRPS}$-values obtained from samples of normal (NC) and dysplastic cervix (DYS) and carcinoma-*in-situ* (CIS). $Q_{TRPS}$ values of samples with normal and apparently normal epithelium (×), dysplasia (△) and carcinoma-*in-situ* (○) respectively.

**Figure 3** Distribution of the $Q_{PSH/TRPS}$ values, obtained from samples of normal cervix (NC) and apparently normal tissue from patients with dysplasia (DYS) or carcinoma-*in-situ*. (CIS).

corresponding area (see Araki *et al.*, 1982). Secondly, instead of protein, another constituent (such as TRPS) could be measured in a serial section, and the ratio of $PSH_r/TRPS$ can then be expected to compensate to a large extent for the variations in protein content from one patient to another but not for individual variations in section thickness. Thirdly, by making a ratio of epithelium: stroma on the same section, such as the $Q_{PSH}$ calculation, a similar type of compensation can be achieved. In fact, it can be seen from Table I that the s.e.m. values for $Q_{PSH}$ are considerably smaller (as a percentage of the corresponding mean; range 4–9%) than for the epithelial and stromal values separately. Although these corrections help to minimise variability on a day to day basis, and between patients, it appears clear to us that the solution to be sought is a double staining method applicable to each individual section.

In cells of a particular type and location we may expect to find *a priori* considerable variations in the SH:disulphide ratio in different physiological and pathological conditions; this ratio will reflect, at

**Figure 4** Distribution of the $Q_{PSH/TRPS}$ values, obtained from samples of normal (NC), dysplastic cervix (DYS) and carcinoma-*in-situ* (CIS). The abscissa ($Q_{PSH/TRPS}$ values) has been expanded compared with **Figures 1–3** to allow better visualisation of the low degree of overlap between the normal population and the pathological situations.

least in part, the redox status of the cell at each time under study. The sum of protein thiols and reactive disulphides may not show significant changes, however, under such conditions as it represents the total reactive protein bound sulphur groups. In fact, as can be seen in Table I, the values found for TRPS of epithelium by the method used here do not vary significantly amongst the various groups studied, indicating a reasonable constancy of this measure in dysplasias and carcinomas-*in-situ* compared to normal. However, the relative constancy of the TRPS values in stroma should be also viewed against the changes in $PSH_r$ previously discussed. Such a comparison suggests that in the stroma adjacent to ANS-Epi, DYS-Epi and CIS there are decreases in reactive disulphides as indicated by the (TRPS-$PSH_r$) values. Indeed, these differences are statistically significant for the stromal values in Groups 2a, 2b, 3a and 3b. For a discussion of the interaction of epithelium and stroma in uterine cervix see Bajardi *et al.*, (1983*b*).

As mentioned above, the ratio of $PSH_r$ to TRPS was expected to compensate at least partially for variability between protein content, and to decrease the s.e.m. values relative to the means; it can be seen in Table I that in fact this does occur. Moreover, the ratio of $PSH_r$:TRPS emphasises the disturbed biochemical events that have occurred in dysplasias and carcinoma-*in-situ* compared to normal – the $Q_{PSH/TRPS}$ depressions are similar at first sight to the depressions previously discussed for $Q_{PSH}$ but the much smaller variability between samples in $Q_{PSH/TRPS}$ in comparison to $Q_{PSH}$, produces a clear discrimination of the individual measures of normal samples from the individual measures of dysplasia and of carcinoma-*in-situ* (Figure 4).

It can also be seen from the results in Table I and Figure 3 for $Q_{PSH/TRPS}$ (and, to a lesser extent for $Q_{PSH}$) that similar changes were observed in the apparently normal epithelium and stroma around a lesion as in the dysplastic or carcinoma-*in-situ* lesions themselves. One possible cause of this unexpected finding is a diffusion out from the lesion of a substance that changes the thiol balance in neighbouring 'normal' cells: the cells of the lesion would thereby exert a field-like effect on adjacent, otherwise normal, cells. A second possibility, and one we favour, is that the area around and containing the lesion has undergone some metabolic change generally, as indicated here by the disturbed $Q_{PSH}$ and $Q_{PSH/TRPS}$ values, but part of this area (the lesion itself) has undergone some additional disturbance that is manifested histologically and clinically as a dysplasia or carcinoma-*in-situ*.

Other references that may be consulted in relation to possible diffuse changes in samples of cervix obtained from patients with carcinoma-*in-situ* or invasive cancer are by Benedetto *et al.*, (1981); Millett *et al.* (1982); and Nöhammer *et al.* (1984).

It is possible that the changes reported here on $PSH_r$ and TRPS are associated with consequences of prior virus infection, for example by herpes or papilloma viruses. There is much current interest in the possible role of such viruses in the development of cancer of the cervix (McBrien & Slater, 1984; Auralian *et al.*, 1981; Fu *et al.*, 1983 and Crum *et al.*, 1984), and we are now studying the effects of such virus infections on $PSH_r$ and the redox status of human cervix samples.

We are grateful to the National Foundation for Cancer Research for financial support and making this collaborative study possible.

### References

ARAKI, T., NAKAE, Y., CHIKAMORI, K., KATSURA, S. & YAMADA, M.-O. (1982). Micro-assay of sulfhydryl group content per tissue protein by tridensitrometry. *Cell. Mol. Biol.*, **28**, 213.

AURALIAN, L., KESSLER, I.I., ROSENSHEIN, N.B. & BARBOUR, G. (1981). Viruses and gynecological cancers: herpes virus protein (ICP10/AG-4), a cervical tumour antigen that fulfils the criteria for a marker of carcinogenicity. *Cancer*, **48**, 455.

BAJARDI, F., BENEDETTO, C., NÖHAMMER, G., SCHAUENSTEIN, E. & SLATER, T.F. (1983*a*). Histophotometrical investigation on the content of protein and protein thiols in the epithelium and stroma of the human uterine cervix. II. Intraepithelial neoplasias. *Histochemistry*, **78**, 95.

BAJARDI, F., JÜTTNER, F. & SMOLLE, J. (1983*b*) Korrespondierende Verhaltensweisen von Epithel und Stroma der Cervix uteri. *Zentbl. Gynakol*, **105**, 257.

BARRON, E.S.G. (1951). Thiol groups of biological importance. *Adv. Enzymol.*, **11**, 201.

BENEDETTO, C., BOCCI, A., DIANZANI, M.U. and 4 others (1981). Electron spin resonance studies on normal human uterus and cervix, and on benign and malignant uterine tumours. *Cancer Res.*, **41**, 2936.

BENEDETTO, C., BAJARDI, F., NÖHAMMER, G., ROJANAPO, W., SCHAUENSTEIN, E. & SLATER, T.F. (1986), (In preparation).

CRUM, C.C., IKENBERG, H. & RICHART, R.M. (1984). Human papilloma virus type 16 and early cervical neoplasia. *New Engl. J. Med.*, **310**, 880.

FRIEDMAN, M. (1973). *The Chemistry and Biochemistry of the Sulphydryl Group in Amino Acids, Peptides and Proteins*. Pergamon Press, Oxford.

FU, Y.S., REAGAN, J.W. & RICHART, R.M. (1983). Precursors of cervical cancer. *Cancer Surveys*, **2**, 359.

HARINGTON, J.S. (1967). The sulphydryl group and carcinogenesis. *Adv. Cancer Res.*, **10**, 247.

JOCELYN, P.C. (1972). *Biochemistry of the SH-Group*. Academic Press, London.

KNOCK, F.E., GOLT, R.M. & OESTER, Y.T. (1967). Protein-sulfydryl groups in cellular control mechanisms and cancer. *J. Amer. Geriat. Soc.*, **15**, 882.

KOSOWER, N.S. & KOSOWER, E.M. (1978). The glutathione status of cells. *Int. Rev. Cytol.*, **54**, 109.

McBRIEN, D.C.H. & SLATER, T.F., (eds.) (1984). *Cancer of the Uterine Cervix. Biochemical and Clinical Aspects*. Academic Press, London.

MANNERVIK, B. & AXELSSON, K. (1980). Role of cytoplasmic thioltransferase in cellular regulation by thiol-disulfide interchange. *Biochem. J.*, **190**, 125.

MILLETT, J.A., HUSAIN, O.A.N., BITENSKY, L. & CHAYEN, J. (1982). Feulgen-hydrolysis profiles in cells exfoliated from the cervix uteri: a potential aid in the diagnosis of malignancy. *J. Clin. Pathol.*, **35**, 345.

NÖHAMMER, G. (1982). Quantitative microspectrophotometrical determination of protein thiols and disulfides with 2,2′-dihydroxy-6,6′-dinaphthyl-disulfide (DDD). The variety of DDD-staining methods demonstrated on Ehrlich ascites tumour cells. *Histochemistry*, **75**, 219.

NÖHAMMER, G., BAJARDI, F., BENEDETTO, C., SCHAUENSTEIN, E. & SLATER, T.F. (1984). Studies on the relationship between epithelium and stroma in sections of human uterine cervix in different pathological conditions. In *Cancer in the Uterine Cervix. Biochemical and Clinical Aspects*, McBrien, D.C.H. & Slater, T.F. (eds) p. 205. Academic Press: London.

RAPKINE, L. (1930). Sur les processus chimique au cours de la division cellulaire. *C.r. Rend. Acad. Sci.*, **191**, 871.

SCHAUENSTEIN, E., GOLLES, J., WALTERSDORFER, H. & SCHAUR, R.J. (1978). Association between the doubling time of various cells and tissues, and the SH-content of their soluble proteins. *Z. Naturforsch.*, **33c**, 79.

SCHAUENSTEIN, E., BAJARDI, F., BENEDETTO, C., NÖHAMMER, G. & SLATER, T.F. (1983). Histophotometrical investigations on the contents of protein and protein thiols of the epithelium and stroma of human cervix. I. Cases with no apparent neoplastic alterations of the epithelium. *Histochemistry*, **77**, 465.

SCOVASSI, A.I., PLEVANI, P. & BERTAZZONI, U. (1983). Eukaryotic DNA polymerases. In *DNA makes RNA makes Protein*, Hunt, T. *et al.* (eds) p. 30. Elsevier Biomedical: Amsterdam.

SHERBERT, G.V. (1983). The cell surface sulphydryl content of metastatic variants of B.16 murine melanoma. *Exp. Cell Biol.*, **51**, 140.

SLATER, T.F., BAJARDI, F., BENEDETTO, C. and 7 others (1985). Protein thiols in normal and neoplastic human uterine cervix. *FEBS Letters*, **187**, 267.

ZIEGLER, D.M., DUFFEL, M.W. & POULSON, L.L. (1980). Studies on the nature and regulation of the cellular thiol:disulphide potential. In *Ciba Foundation Symposium No. 72* Elliott K. & Whelan, J. (eds), p. 191. Excerpta Medica: Amsterdam.